# An Enigmatic Elevation of Troponin in a Patient With Seizures

**DOI:** 10.7759/cureus.12635

**Published:** 2021-01-11

**Authors:** Harini G Lakshman, Pal Satyajit Singh Athwal, Anoosha Ponnapalli, Syed Ahmed, Nimit Dalal

**Affiliations:** 1 Internal Medicine, Michigan State University/Hurley Medical Center, Flint, USA; 2 Cardiovascular Division, University of Minnesota, Minneapolis, USA; 3 Department of Infectious Diseases, University of Washington Valley Medical Center, Renton, USA; 4 Cardiology, Michigan State University/Hurley Medical Center, Flint, USA; 5 Internal Medicine, Trumbull Regional Medical Center, Warren, USA

**Keywords:** false negative troponin, post seizures elevated troponin

## Abstract

A 64-year-old African American male, with past medical history of hypertension, depression, and seizure disorder, presented with an episode of generalized tonic-clonic seizure. He was treated for seizures, and after 48 hours seizure-free, the patient started complaining of chest tightness and troponin levels were found to be 34.71 ng/mL. No evidence of myocardial infarction was found after extensive diagnostic workup, including cardiac catheterization. We suspect alternative causes of elevated troponin including post-seizure and transient takosubo cardiomyopathy.

## Introduction

Markers of cardiac injury are used to diagnose myocardial infarction (MI) but problems arise in cases of falsely elevated markers leading to unnecessary interventions. Cardiac markers evolved from aspartate aminotransferase (AST) to lactate dehydrogenase (LDH), creatine kinase (CK), CK-MB, and finally to troponins. Troponins are considered the most sensitive and specific markers and are composed of three isoforms: troponin T, troponin I, and troponin C. To diagnose MI, cardiac troponin should rise and/or fall above the 99th percentile combined with evidence of myocardial ischemia as suggested by one of the following:

1) symptoms of myocardial ischemia;

2) new ischemic electrocardiographic changes;

3) new pathological Q waves;

4) new ischemic regional wall motion abnormalities on cardiac imaging;

5) acute coronary thrombus on coronary angiography.

Causes of falsely elevated troponins should be ruled out if no myocardial infarction is present. We report a case of very significantly raised troponins following an epileptic episode. Though seizures are a known cause of raised troponin levels, such significantly high levels increase suspicion of stress-induced cardiomyopathy during an epileptic episode.

## Case presentation

A 64-year-old African American male was admitted for an episode of seizure. His seizure was treated with levetiracetam. A CT scan of his head was done since the patient had a fall during a seizure, which was negative for any acute hemorrhage or focal lesions. After 48 hours seizure-free, the patient started complaining of chest tightness. It was not associated with any diaphoresis, palpitation, nausea, vomiting, or shortness of breath. On examination, his vital signs were stable with a heart rate of 82, blood pressure of 122/82 mmHg, respiratory rate of 18, with oxygen saturation of 98% at room air. He was alert and oriented. Lungs were clear to auscultation. Cardiovascular examination revealed normal S1, S2 with regular rate and rhythm. Neurological and abdominal examinations were normal.

Past medical history

Past medical history was significant for hypertension treated with amlodipine, seizure disorder on valproate and levetiracetam, and depression on sertraline 20mg.

Differential diagnosis

A 64-year-old male with hypertension and chest tightness raises suspicion for myocardial infarction which is very important to be ruled out immediately. Raised levels of cardiac troponin further make MI likely. Other possible differential includes aortic dissection and pulmonary embolism.

Investigations

Cardiac troponin along with electrocardiography was ordered initially. Troponin was found to be significantly raised as shown in Table 1.

**Table 1:** Troponin levels.

Troponin Levels (ng/ml):	
09/01/2020	12.57
09/01/2020	34.71
09/02/2020	49.64
09/02/2020	40.37
09/02/2020	47.27
09/02/2020	35.5

**Figure 1:**
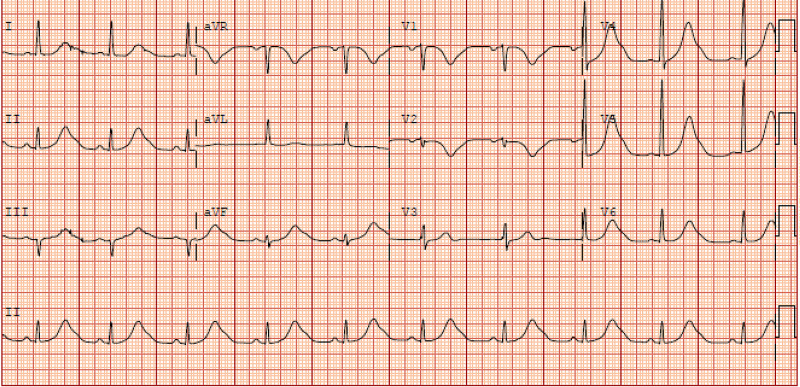
ECG showed hyperacute T waves and U waves

Electrocardiogram (ECG) showed hyperacute T waves and U waves (Figure 1).

**Figure 2:**
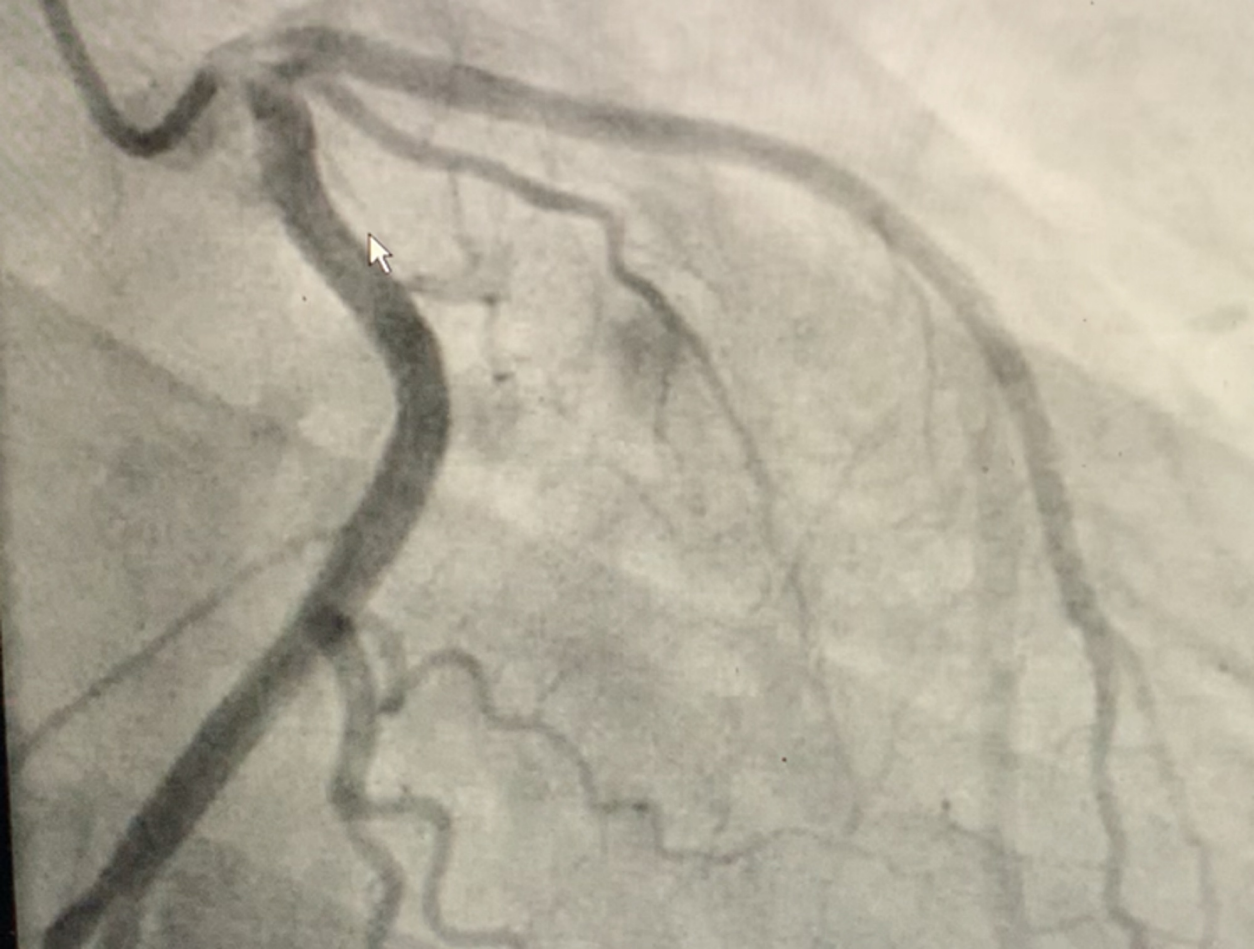
Coronary angiography revealed disease free arteries without any stenosis.

Cardiac catheterization was done due to significantly raised troponin levels, with suspicion of non-ST-elevation myocardial infarction (NSTEMI). Coronary angiography revealed disease-free arteries without any stenosis (Figure 2).

Echocardiography was performed which showed a normal ejection fraction of 50-55%. Chest CT with contrast was done to rule out PE which was normal.

Management

The patient was initially treated with loading doses of aspirin and ticagrelor, heparin for acute coronary syndrome (ACS) protocol was started. He was given nitroglycerin sublingually for chest pain, which helped decrease the pain. Heparin was continued for 24 hrs and since the cardiac catheterization was normal and the troponin trended down, it was discontinued.

Follow up

The patient was given supportive therapy after cardiac catheterization. His aspirin, ticagrelor, and heparin were discontinued and he was treated for his seizures with loading doses of Keppra and his troponin trended down to 35.5 ng/ml. He was discharged home symptom-free and was followed up after a week in the adult clinic. His troponin was back to <0.01 ng/ml and he did not complain of any symptoms.

## Discussion

This case presentation is an example of the fact that elevated troponin is not limited to cardiovascular pathology but a broad differential should be considered. Cardiac troponin (cTn) is a universally accepted marker for cardiac injury and plays a crucial role in the diagnosis of acute myocardial infarction. These markers of cardiac injury are regulatory proteins controlling the interactions between actin and myosin mediated by calcium [1]. The history of markers for myocardial damage has evolved with time. CK, aspartate aminotransferase, and lactate dehydrogenase were the initial markers used, which were nonspecific to myocardial injury. Later on, measurement of cTnT and cTnI became the standard to detect myocardial damage, which is considered very highly specific [2]. Cardiac markers gradually increase with myocardial hypoperfusion as it is one of the major criteria to diagnose MI according to the Fourth Universal Definition of Myocardial Infarction [3]. Raised troponin levels as in this scenario further require cardiac catheterization as both a diagnostic as well as a therapeutic step. Such an invasive procedure is lifesaving but consideration should be given to other causes if there is a higher suspicion for non-cardiac causes. Causes of false-positive elevation of troponin can be divided into cardiac and non-cardiac causes (Table 2) [4,5].

**Table 2:** Causes of Raised Troponin Levels. ACS: acute coronary syndrome

Causes of Raised Troponin Level	
Non- ACS Cardiac causes	Non-Cardiac causes
Heart failure	Heterophile antibodies
Myopathies	Human anti-mouse antibodies
Cardiac contusion	Autoantibodies
Takotsubo cardiomyopathy	Malfunction of analyzer
Endomyocardial Biopsy	High Alkaline phosphatase
Aortic dissection	Interference by bilirubin, hemoglobin, lipidemia
Arrhythmia (both bradyarrhythmia and tachyarrhythmia)	Renal failure
Cardiotoxic drugs	Pulmonary embolism
Cardioversion	Sepsis
Stenting/ Angioplasty	Burns
	Stroke

Brobbey et al. reported the first case of raised troponin after a grand mal seizure without any evidence of cardiac pathology. Value of troponins ranged between 5.5 ng/mL and 6.3 ng/mL, in contrast to our case in which troponins were very highly elevated from 12.57 to 49.64 ng/mL [6]. A study of 14 patients with cardiovascular disease risk factors also demonstrated some correlation between seizures and raised troponins levels [7]. The pathophysiology remains obscure but there might be other underlying causes of raised troponin which were not ruled out. “Reversible ischemia model” is believed to be a hypothesis behind raised troponin levels in seizures, which are temporarily reduced coronary blood flow [8].

Falsely elevated troponins could be due to technical errors or from other causes discussed above, so other cardiac biomarkers should be ordered along with troponins to have more evidence for an invasive procedure. In the case of falsely elevated troponin, different causes should be ruled out using appropriate investigations. In our case history, examination, lab workup, and imaging revealed no evidence of any potential cause except the fact that the patient had an episode of seizure.

Stress-induced cardiomyopathy, also known as Takotsubo cardiomyopathy, is also one of the causes that could account for this elevation of troponin. It is characterized by transient dysfunction of the apical portion of the left ventricle with compensatory hyperkinesia of the basal walls, thus resulting in apical ballooning that gives the heart the appearance of a Japanese octopus's trap or Takotsubo. It is triggered by emotional or physical stress and is very similar to acute coronary syndrome, although the coronary arteries are normal. Usually, cardiac enzymes are slightly increased, and serial electrocardiographic changes often consist of ST-segment elevation followed by inversion of T waves [9]. Although the cause of Takotsubo cardiomyopathy is unknown, exaggerated sympathetic stimulation has been proposed as a central factor in the pathophysiology. Although in our case apical ballooning was not seen, given the patient had a seizure which could be the stress factor that caused elevation of troponins, as the ballooning might always not be present during the ventriculography. The only reproducibility is by acetylcholine or ergonovine-induced vasospasm or apical dyskinesia, which was not done due to the non-availability of these tests.

## Conclusions

The decision of whether or not to perform a life-saving invasive procedure in a patient with nonspecific elevation of troponin remains very challenging. Coronary catheterization was normal and probably had decreased utilization in our case but it was a crucial step to rule out MI in the setting of chest pain and raised troponin levels. Through this case we want to highlight the importance of false-positive troponin elevation and possible association with an episode of seizure or transient stress-induced cardiomyopathy.
